# A feasibility double-blind trial of levothyroxine vs. levothyroxine-liothyronine in postsurgical hypothyroidism

**DOI:** 10.3389/fendo.2025.1522753

**Published:** 2025-03-10

**Authors:** Giao Q. Phan, Sahzene Yavuz, Angeliki M. Stamatouli, Ritu Madan, Shanshan Chen, Amelia C. Grover, Naris Nilubol, Pablo Bedoya, Cory Trankle, Roshanak Markley, Antonio Abbate, Francesco S. Celi

**Affiliations:** ^1^ Division of Surgical Oncology, Neag Cancer Center, UConn Health, Farmington, CT, United States; ^2^ Department of Medicine, The Guthrie Clinic, Sayre, PA, United States; ^3^ Division of Endocrinology, Diabetes and Metabolism, Virginia Commonwealth University, Richmond, VA, United States; ^4^ Division of Surgical Oncology, Virginia Commonwealth University, Richmond, VA, United States; ^5^ Surgical Oncology Program, National Cancer Institute, National Institutes of Health, Bethesda, MD, United States; ^6^ Virginia Diabetes and Endocrinology, Richmond, VA, United States; ^7^ Division of Cardiology, Virginia Commonwealth University, Richmond, VA, United States; ^8^ Berne Cardiovascular Research Center, University of Virginia, Charlottesville, VA, United States; ^9^ Department of Medicine, UConn Health, Farmington, CT, United States

**Keywords:** hypothyroidism, post-surgical hypothyroidism, combination therapy, clinical trial, levothyroxine, liothyronine

## Abstract

**Context:**

Despite normalization of Thyrotropin (TSH), some patients with hypothyroidism treated with Levothyroxine (LT4) report residual symptoms which may be attributable to loss of endogenous triiodothyronine (T3).

**Objective:**

Feasibility trial LT4/liothyronine (LT3) combination vs. LT4/placebo in post-surgical hypothyroidism.

**Design:**

Double-blind, placebo-controlled, 24-week study.

**Setting:**

Academic medical center

**Patients:**

Individuals with indications for total thyroidectomy and replacement therapy.

**Interventions:**

LT4/LT3 5 mcg (twice daily) vs. LT4/placebo (twice daily). LT4 was adjusted at 6- and 12-weeks with the goal of baseline TSH ± 0.5 mcIU/ml.

**Main Outcome Measures:**

Changes in body weight, cholesterol, TSH, total T3, free tetraiodothyronine (T4). Cardiovascular function, energy expenditure, and quality of life (ThyPRO-39) were assessed in patients who completed at least the 3-month visit, last measure carried-forward.

**Results:**

Twelve patients (10 women and 2 men), age 51 ± 13.8 years (7 LT4/placebo, 5 LT4/LT3), were analyzed. No significant differences were observed in TSH. Following thyroidectomy, LT4/placebo resulted in higher free T4 + 0.26 ± 0.15 p<0.005 and lower total T3 -18 ± 9.6 ng/dl p<0.003, respectively, not observed in the LT4/LT3 group. The LT4/placebo group had a non-significant increase in body weight, +1.7 ± 3.8 Kg, total- and LDL-cholesterol +43.1 ± 72.8 and +32.0 ± 64.4 mg/dl. Conversely the LT4/LT3 group changes were -0.6 ± 1.9 Kg, -28.8 ± 49.0 and -19.0 ± 28.3 mg/dl, respectively, all non-significant. Non-significant improvement were observed in ThyPRO-39 measures in both groups, while energy expenditure, and diastolic function increased in the LT4/LT3 group.

**Conclusions:**

In this group of patients with post-surgical hypothyroidism LT4 replacement alone does not normalize free T4 and total T3 levels and is associated with non-significant increase in weight and cholesterol. LT4/LT3 combination therapy appears to prevent these changes.

**Clinical Trial Registration:**

Clinicatrials.gov, identifier NCT05682482.

## Introduction

The treatment of hypothyroidism is based on the substitution of synthetic T4, levothyroxine (LT4), for the loss of endogenous thyroid hormone (TH) production, and its efficacy is measured by the normalization of thyrotropin (TSH) (1). This strategy assumes that pituitary euthyroidism indicates restoration of hormonal signaling to all tissues targeted by TH action. While most patients do well on LT4 alone, a sizable minority, in excess of 40% in a study (2), reports residual symptoms consistent with hypothyroidism, which may be attributed to the loss of endogenous production of T3 not completely compensated by the peripheral conversion of exogenous T4 into T3 (3, 4). Studies conducted in animal models of hypothyroidism demonstrated that LT4 alone is not sufficient to restore T3 and T4 concentrations in all tissues, while the combination of LT4/liothyronine (synthetic T3, LT3) can (5–7). While a prospective study indicated that LT4 therapy is able to restore circulating levels of T3 (8), previous observations and large longitudinal studies reported that effective (*i.e.* resulting in TSH normalization) LT4 therapy is associated with decrease in T3 and increase in free T4 (9–11). Several trials were conducted to test the effects of T3-containing therapies (12–25). The results have been inconclusive because of a lack of statistical power, heterogeneous populations and treatment schemes (26). While professional organizations’ guidelines do not support the use of T3-containing therapies on a routine basis (1, 27–29), they lament the lack of evidence and encourage the development of well-designed studies to assess the efficacy of these treatment options (30).

Patients undergoing total thyroidectomy are unique since they transition from a state of euthyroidism to complete dependence from exogenous administration of TH. To this end, these patients represent an ideal experimental model to assess the effects of different modalities of replacement therapy on circulating TH concentrations and end-organ effects of TH action.

Here we present a proof-of-concept/feasibility study of LT4/placebo vs. LT4/LT3 replacement therapy in patients undergoing total thyroidectomy. This study was designed to explore the changes in TH and in indices of hormonal action within, and between study groups, with the goal of obtaining point estimates of these measures to adequately power subsequent large trial(s).

## Materials and methods

### Study design

**Figure 1:**
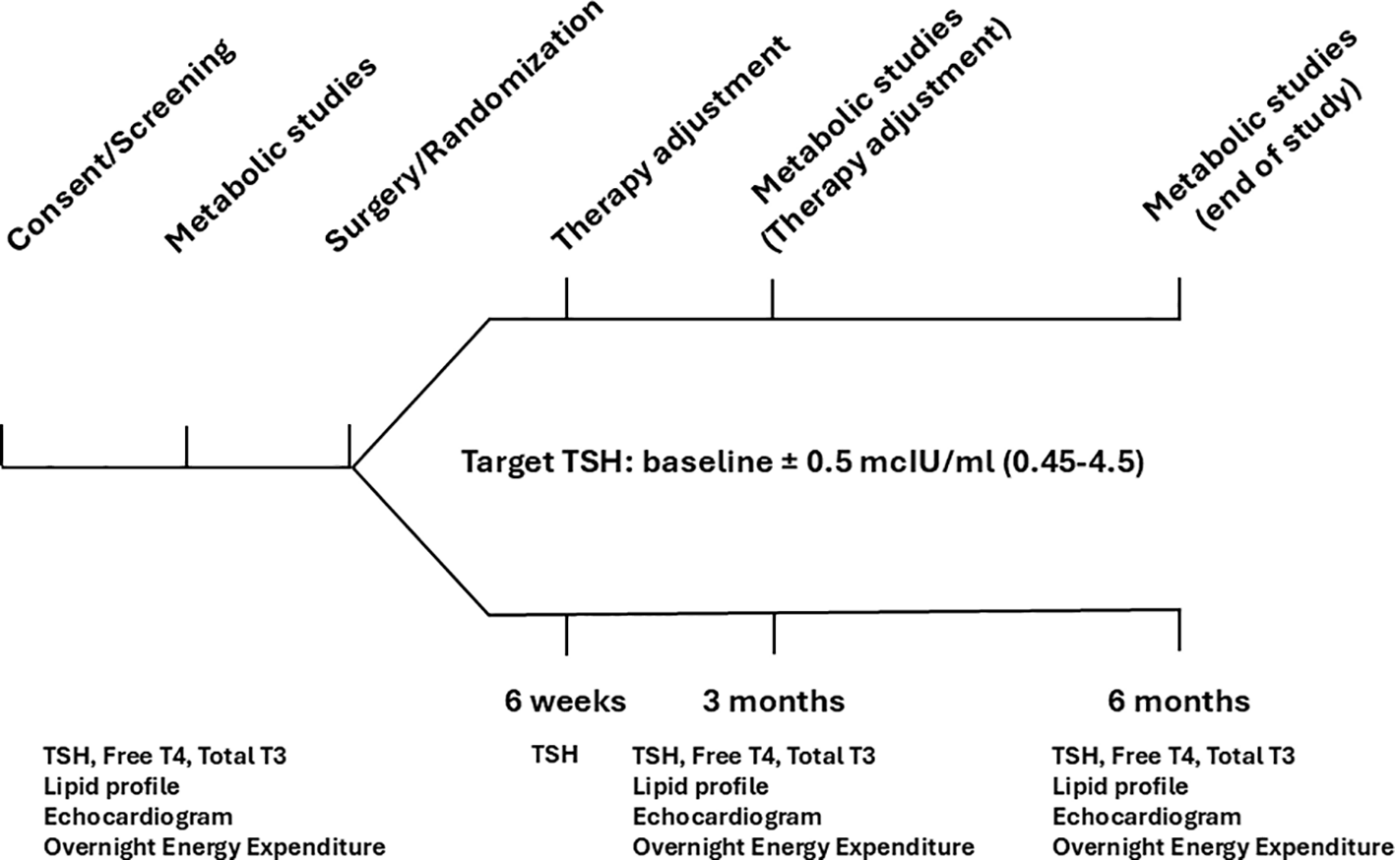
Study scheme and procedures.

This was a double-blind, placebo-controlled, two active comparators (LT4/placebo vs. LT4/LT3) parallel, six-month study (**Figure 1**) in patients undergoing thyroidectomy designed to obtain point estimates of the effect size of each intervention. The study was approved by the Virginia Commonwealth University IRB, and all study participants provided written informed consent (Clinicaltrials.gov ID NCT04782856). The research was completed in accordance with the Declaration of Helsinki as revised in 2013.

### Study participants, inclusion criteria, and randomization

Inclusion criteria were age>18 years, normal TSH, and clinical indication for total thyroidectomy. Exclusion criteria were indication for TSH suppression; history of hypothyroidism or thyrotoxicosis; congestive heart failure or unstable coronary artery disease (angina, coronary event, or revascularization within 6 months); atrial fibrillation; uncontrolled hypertension (>140/90 mmHg at screening); uncontrolled diabetes (HbA1c>8% at screening); pregnancy, breastfeeding, or planned pregnancy during the study; history of major depression or psychosis; use of drugs known to interfere with TH absorption or activity (e.g. antiacids, bile acid sequestrants, dopaminergic or dopamine antagonists, amiodarone) (31); conditions that in the opinion of the principal investigator may impede the successful completion of the study. Participants taking lipid-lowering medications were instructed not to change their regimen during the trial. Following enrollment, the participants were randomized to the treatment groups by the investigational pharmacy.

### Sample size

This feasibility study was designed to obtain point estimates of the effects of LT4/LT3 compared to LT4/placebo on weight, and LDL cholesterol. Based on prior observations (32, 33), with a sample size of 15 participants per arm, a difference of 2.2 Kg would provide 80% power at a significance of 0.05. Similarly, a difference in LDL cholesterol of 8% would provide 70% power at a significance of 0.05. These estimates were based on the assumption of 50% of the effect size observed in our prior crossover LT3 substitution trial (32) and an expected 40% increase in average serum total T3 following LT4/LT3 combination therapy compared to LT4 alone (33). Changes in quality of life (ThyPRO-39) (34), cardiovascular parameters and energy expenditure were considered exploratory endpoints. Due to the exploratory nature of the study the accrual target of the study was placed at 30 participants.

### Screening visit

This encounter was conducted to verify inclusion and exclusion criteria, and to allow participants to provide informed consent.

### Baseline visit

Study volunteers were admitted prior to surgery (usually the night before the procedure) for overnight energy expenditure recording in a whole-room indirect calorimeter (35, 36). The next morning, study participants underwent a standard transthoracic Doppler echocardiogram to measure left ventricular dimensions and diastolic/systolic function (37), including the myocardial performance (Tei index) obtained by subtracting the ejection time (ET) from the interval between cessation and onset of the mitral inflow velocity to give the sum of isovolumetric contraction time (ICT) and isovolumentric relaxation time (IRT) and calculated as (ICT+IRT)/ET, with smaller numbers reflecting better left ventricular diastolic and systolic function (38); Arterial elastance (Ea) was measured as left ventricular end-systolic pressure (LVESP), estimated as 0.9 x systolic blood pressure divided by LV stroke volume, and end-systolic elastance (EES) measured as LVESP divided by the left ventricular end-systolic volume, and expressing Ea/EES as a measure of ventricular-arterial coupling (39).

Following the echocardiogram, anthropometric measurements and quality of life assessment by ThyPRO-39 (34) were recorded, and fasting blood sampling for TSH, free T4, total T3, and lipid panel was collected. At the time of discharge from surgery (usually in the evening of the same day), study participants were given two study medications bottles, one “AM”, containing LT4/placebo or LT4/LT3, and a second “PM” containing placebo or LT3 (*see study medications and therapy adjustments*).

### Six-week visit

Six weeks following surgery, study participants were seen for therapy adjustment which consisted of a brief exam, and blood draw for TSH measurement. Study medications were then adjusted and delivered to the participants (*see below*).

### Three- and six-month visits

Three- and six-months following surgery, study participants returned for an overnight visit. The procedures were identical to the baseline visit, apart from therapy adjustment at the three-month visit. Upon study completion, patients returned to the care of their endocrinologists.

### Study medications

Patients were randomized to (a) LT4/placebo, starting at a dose of 1.6 mcg/Kg (32, 40), or (b) LT4/LT3 combination therapy. For the LT4/LT3 arm, the initial dose was calculated by decreasing the estimated dose LT4 by 25 mcg, and adding a fixed dose of LT3, 5 mcg twice daily according to our pharmacokinetics modeling (33). Study drugs were over-encapsulated in identical capsules as LT4/placebo or LT4/LT3 (AM), and placebo or LT3 (PM). The LT4 dose was adjusted at the six-week and three-month visits using the scheme reported in **Table 1** by an unblinded physician (SY, AMS, RM) and delivered by courier; no changes were made to the LT3 dose throughout the study. Study participants were instructed to take the AM capsule in the morning, with an empty stomach, to wait at least 30 minutes before having breakfast or taking any other medications, and to take the PM dose at least 30 minutes before dinner. During the whole-room indirect calorimetry measurements, study volunteers were instructed to take their study medications following their regular schedule.

**Table 1:** Levothyroxine adjustment scheme.

TSH (mcIU/ml)	Dose adjustment
Baseline ± 0.5	No change
Baseline > 0.5 -7.0	Increase by 10%
> 7.0	Increase by 20%
Baseline < 0.5 - 0.1	Decrease by 10%
< 0.1	Decrease by 20%

Dose adjustments were common to both treatments, therapy rounded to the closest available dose.

### Statistical analysis

Two-tailed unpaired t test was used to compare data between treatment arms, while two-tailed paired t test was used to compare baseline vs. end-of-study results within the same treatment arm. Results are expressed as mean ± SD and median and interquartile ranges (IQR); p < 0.05 was considered the threshold for significance. Analyses were conducted using Prism version 5 (GraphPad, La Jolla, CA) and SPSS Version 29.0 (IBM, Chicago, IL) in participants who completed at least the three-month visit, with last measure carried forward. No adjustment for multiple comparisons was made.

## Results

### Study participants

**Figure 2:**
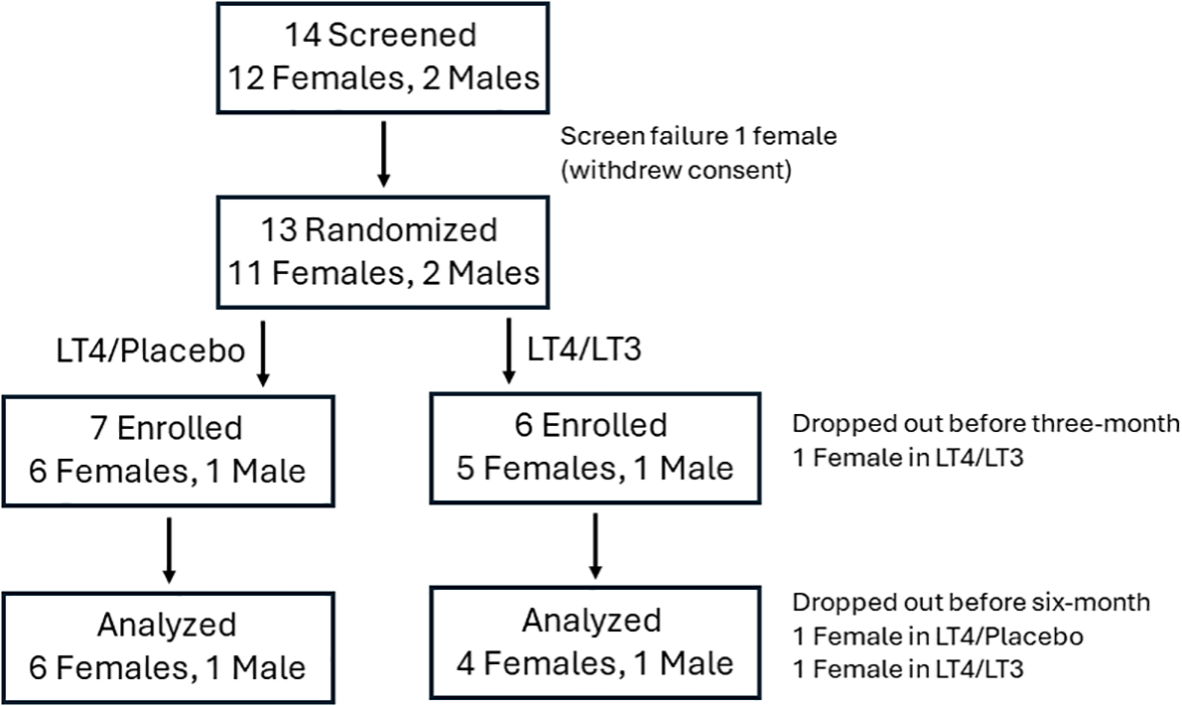
CONSORT chart. The analysis was conducted (last measure carried forward) on study participants who completed the three-month visit.

Thirteen participants (11 females, 2 males, age 51 ± 13.2 years) were randomized between 10/29/2020 and 10/26/2022; twelve participants who completed at least the three-month follow up visit were included in the analysis. Of them, five were allocated to LT4/LT3, and seven to LT4/placebo; their characteristics are reported in **Table 2**. Screening, randomization, drop-out and completion of the study data are reported in **Figure 2**. No patient-reported adverse event was recorded. A change in dosing outside the titration scheme (dose reduction) was deemed necessary because of sustained TSH suppression at month-3 in a LT4/LT3 group patient. This was attributed to oral GLP-1 analog therapy (41).

**Table 2:** Baseline characteristics of study participants included in the analysis.

	All participants	LT4/LT3	LT4/placebo	Significance
Number (sex)	12 (10 f, 2 M)	5 (4F, 1M)	7 (6F, 1M)	n/a
Age (years)	51.7±13.8	50.0±13.8	53.0±13.6	P=0.728
Weight (Kg)	81.8±20.2	90. 0±23.8	76.0±16.7	P=0.257
Ethnicity	9 Caucasian, 3 Black	3 Caucasian, 2 Black	6 Caucasian, 1 Black	n/a
Multinodular goiter	6	3	3	n/a
Papillary thyroid cancer	5	1	4	n/a
Medullary thyroid cancer	1	1	0	n/a

### Study medications dose adjustments

The average initial LT4 dose in the LT4/placebo group was 119.3 ± 24.8 mcg (1.57 ± 0.0 mcg/Kg), while at end-of-study it was 107 ± 23.5 mcg (1.43 ± 0.4 mcg/Kg) (p=0.406). The LT4 dose was unchanged in one patient, increased in one, and decreased in four. In the LT4/LT3 group the initial average LT4 dose was 120.00 ± 41.1 mcg (1.31 ± 0.1 mcg/Kg), while at end-of-study it was 86.5 ± 10.1 mcg (1.03 ± 0.2 mcg/Kg) (p=0.023). The LT4 dose was increased in one patient and decreased in four. The data are ported in **Table 2**.

### Thyroid hormone and TSH

Compared to baseline (pre-thyroidectomy) TSH, in the LT4/placebo group no differences were observed at end-of-study (1.64 ± 0.70 vs. 1.64 ± 1.09 mcIU/ml), while a non-significant decrease (1.57 ± 0.63 vs. 0.86 ± 1.46 mcIU/ml, p=0.265) was observed in the LT4/LT3 group. At end-of-study the free T4 concentrations were significantly increased in the LT4/placebo (0.91 ± 0.12 vs. 1.17 ± 0.26 ng/dl, p=0.005), while no significant changes (0.96 ± 0.13 vs. 1.02 ± 0.26 ng/dl, p=0.645) were observed in the LT4/LT3 group. The total T3 concentrations were significantly decreased in the LT4/placebo (98.7 ± 10.9 vs. 80.7 ± 14.6 ng/dl, p=0.003), while a non-significant increase (96.8 ± 15.7 vs. 121.4 ± 23.9 ng/dl, p=0.142) was observed in the LT4/LT3 group. Similarly, at end-of-study the total T3/free T4 ratio was significantly decreased in the LT4/placebo (110.0 ± 22.2 vs. 71.0 ± 16.6, p<0.001), while a non-significant increase (103.7 ± 25.9 vs. 121.5 ± 20.0, p=0.142) was observed in the LT4/LT3 group.

In between groups analyses, no significant differences were observed in TSH and free T4 at end-of-study (p=0.337 and p=0.135, respectively), while the differences in total T3 and total T3/free T4 were statistically significant (LT4/Placebo -18.0 ± 9.6 vs. LT4/LT3 20.5 ± 28.8 ng/dl, p=0.005 and LT4/Placebo -39.1 ± 12.1 vs. LT4/LT3 17.8 ± 27.5, p<0.001, respectively). The data are ported in **Table 3**.

**Table 3:** Thyroid hormone, TSH, and study medications.

	LT4/LT3				LT4/placebo				
Parameter	Baseline	End of study	Delta	p	Baseline	End of study	Delta	p	p for Delta
TSH (mcIU/ml)	1.57±0.63	0.86±1.46	-0.59±1.14	0.265	1.64±0.70	1.64±1.09	0.00±1.19	0.998	0.337
FreeT4 (ng/dl)	0.96±0.13	1.02±0.26	0.05±0.24	0.645	0.91±0.12	1.17±0.26	0.26±0.15	**0.005**	0.135
T3 (ng/dl)	96.8±15.7	121.4±23.9	20.5±28.8	0.142	98.7±10.9	80.7±14.6	-18.0±9.6	**0.003**	**0.005**
T3/FreeT4	103.7±25.9	121.5±20.0	17.8±27.5	0.221	110.0±22.2	71.0±16.6	-39.1±12.1	**<0.001**	**<0.001**
LT4 dose (mcg)	120±41.1	86.5±10.1	-33.5±34.6	0.096	119.3±24.8	107.0±23.5	-12.3±22.8	0.406	n/a
LT4 dose (mcg/Kg)	1.31±0.14	1.03±0.22	-0.28±0.18	**0.023**	1.57±0.04	1.43±0.41	-0.14±0.41	0.406	n/a
LT3/LT4	1:9.0±1.4	1:6.8±1.2	-2.2±2.2	**0.003**	n/a	n/a	n/a	n/a	n/a

Bold font indicates statistical significance.

### Lipid parameters

In the LT4/placebo group, compared to baseline, a non-significant increase in total and LDL-cholesterol was observed at end-of-study (213.3 ± 69.2 vs. 256.4 ± 105.6 mg/dl p=0.168, and 131.6 ± 49.3 vs. 163.6 ± 84.1 mg/dl p=0.236, respectively). Conversely in the LT4/LT3 group a non-significant decrease in total and LDL-cholesterol was observed at end-of-study (214.4 ± 48.8 vs. 179.8 ± 20.0 mg/dl p=0.214, and 132.6 ± 37.6 vs. 109.8 ± 15.4 mg/dl p=0.163, respectively). With respect to HDL-cholesterol and triglycerides, in the LT4/placebo group, a non-significant increase in HDL-cholesterol and triglycerides was observed at end-of-study. In the LT4/LT3 group, when compared to baseline, non-significant increase in HDL-cholesterol, and decrease in triglycerides were observed at end-of-study. The data are ported in **Table 4**.

**Table 4:** Lipid parameters, weight and energy expenditure assessment.

	LT4/LT3				LT4/placebo				
Parameter	Baseline	End of study	Delta	p	Baseline	End of study	Delta	p	p for Delta
Total Cholesterol (mg/dl)	214.4±48.8	179.8±20.0	-28.8±49.0	0.214	213.3±69.2	256.4±105.6	43.1±72.8	0.168	0.070
LDL Cholesterol (mg/dl)	132.6± 37.6	109.8± 15.4	-19.0± 28.3	0.163	131.6±49.3	163.6±84.1	32.0±64.4	0.236	0.110
HDL Cholesterol (mg/dl)	53.2±11.2	56.8±16.3	3.0±6.5	0.322	58.4±19.8	63.0±18.6	4.6±12.5	0.373	0.880
Triglycerides (mg/dl)	151.8±135.1	100.0±50.3	-43.2±94.2	0.322	124.0±71.0	150.0±96.3	26.0±38.2	0.122	0.070
Weight (Kg)	90.0±23.8	89.2±23.5	-0.6±1.9	0.457	76.0±16.6	77.7±17.8	1.7±3.8	0.294	0.231
EE (Calorie/24 hour)	1,655.8±362.6	1,683.8±388.9	24.5±29.5	0.147	1,544.4± 241.3	1,504.9±213.9	-39.5±68.2	0.215	**0.03**
RQ	0.87± 0.02	0.88± 0.03	0.01±0.03	0.464	0.85± 0.03	0.89± 0.1	0.04± 0.08	0.327	0.125

Bold font indicates statistical significance.

### Weight and energy metabolism assessment

A non-significant increase in body weight was observed at end-of-study in the LT4/placebo group (76.0 ± 16.6 vs. 77.7 ± 17.8 Kg p=0.294), not seen in the LT4/LT3 group (90.0 ± 23.8 vs. 89.2 ± 23.5 Kg p=0.457). Conversely, compared to baseline, the LT4/placebo group had a non-significant decrease in energy expenditure (1,544.4 ± 241.3 vs. 1,504.9 ± 213.9 calorie/24hours p=0.215) while the LT4/LT3 group showed an opposite trend (1,655.8 ± 362.6 vs. 1,683.8 ± 388.9 mg/dl calorie/24hours p=0.147). When the pre-post changes in energy expenditure were analyzed between groups a significant (p=0.03) difference was observed. The data are ported in **Table 4**.

### Cardiovascular parameters

No significant differences were observed in heart rate, blood pressure and ejection fraction between baseline and end-of-study in both groups. Statistically significant differences were observed between groups in Tei index, an indicator of diastolic function: compared to baseline, the LT4/placebo group showed an increase of +18.2 95% [IQR 3.8, 100], while the LT4/LT3 group showed a decrease of -12.7 95% [IQR -21.7, -8.5], p=0.005, with negative changes reflecting better function (38). The data are ported in **Table 5**.

**Table 5:** Cardiovascular parameters.

	LT4/LT3				LT4/placebo				
	Baseline	End of study	Delta	p	Baseline	End of study	Delta	p	p for Delta
SBP (mmHg)	125[119-131]	128 [120-140]	2.4 [0.4 – 6.5]	0.08	130 [127-137]	127 [171-136]	-8.5 [-10.7 - 0]	0.25	0.11
DBP (mmHg)	75 [63-83]	74 [58-85]	-2.4 [-10.1 – 3.8]	0.42	76 [70-89]	73 [62-84]	-11.4 [-15.8 – 15.2]	0.27	0.53
HR (bpm)	81 [61-100]	70 [61-82]	1.4 [-28.4 – 9.7]	0.69	81 [50-95]	81 [54-96]	1.1 [-2.0 – 16.0]	0.29	0.53
LVEDV (ml)	89 [68-108]	67 [64-108]	-4.0 [-24.9 – 15.5]	0.50	90 [82-98]	75 [75-90]	-5.6 [-23.0 - 0]	0.12	0.76
LVESV (ml)	37 [26-51]	27 [24-44]	-15.1 [-32.2 – 17.0]	0.35	35 [29-37]	29 [26-39]	-6.9 [-14.0 – 2.9]	0.20	0.43
LVEF (%)	59 [54-62]	59 [57-66]	8.0 [-5.7 – 14.6]	0.23	60 [54-70]	64 [57-65]	-3.7 [-7.9 – 5.6]	0.60	0.43
E (ms)	77 [54-85]	74 [51-84]	-1.2 [-12.6 – 7.5]	0.59	71 [56-76]	68 [57-79]	1.8 [-8.1 – 26-1]	0.61	0.64
A (ms)	57 [44-96]	66 [49-89]	0 [-7.0 – 18.4]	0.85	58 [42-93]	65 [55-76]	12.2 [-18.5 – 13.5]	0.74	0.76
E/A ratio	1.1 [0.9-1.3]	1.0 [0.9-1.2]	-2.4 [-19.1 – 6.5]	0.35	1.2 [0.8-1.2]	1.2 [0.9-1.3]	-3.1 [-19.0 – 2.9]	0.40	1
DT (ms)	210 [180-220]	227 [209-271]	8.1 [-2.4 – 50.4]	0.14	190 [155-208]	211 [183-239]	16.8 [-4.1 – 19.5]	0.06	1
E/DT ratio	0.34 [0.26-0.47]	0.30 [0.22-0.36]	-10.7 [-35.4 – 6.7]	0.23	0.39 [0.27-0.43]	0.31 [0.23-0.43]	-12.9 [-22.5 – 34.0]	0.61	1
AE (ms)	419 [370-482]	420 [381-451]	-3.9 [-14.3 – 14.8]	0.89	441 [339-495]	419 [398-478]	3.0 [-13.1 – 17.1]	0.61	0.88
ET (ms)	270 [266-325]	309 [262-324]	1.1 [-8.6 – 14.4]	0.69	348 [259 -353]	308 [264-318]	-0.8 [-10.2 – 12.0]	0.60	0.64
Tei Index	0.36 [0.32 – 0.68]	0.32 [0.29-0.55]	-12.7 [-21.7 - -8.5]	**0.04**	0.27 [0.25-0.40]	0.36 [0.33-0.55]	18.2 [3.8 - 100]	0.08	**0.005**
E’ (cm/s)	8.1 [7.3-11.7]	6.9 [6.5-12.5]	-5.3 [19.9 – 10.8]	0.69	9.1 [9.0 – 9.8]	9.5 [7.6-10.0]	4.9 [-21.3 – 11.1]	1	0.88
E/E’ (cm/s)	8.4 [5.3-11.0]	8.2 [6.1-10.4]	1.8 [-9.0 – 19.4]	0.89	7.2 [5.9-8.3]	7.5[6.1-9.2]	-3.4 [-16.7 – 22.9]	0.87	1
E’/DT	0.04 [0.03-0.06]	0.03 [0.03-0.05]	-15.2 [-39.1 – 5.0]	0.35	0.05 [0.04-0.06]	0.04 [0.03-0.05]	-9.9 [-28.6 – 2.0]	0.09	0.53
Ea	2.1 [2.0-2.9]	2.8 [1.9-2.9]	-1.9 [-12.5 – 30.8]	0.69	2.1 [1.8-2.7]	2.2 [1.8-2.7]	-2.2 [-12.1 – 26.5]	0.87	1
Ees	3.0 [2.4-4.5]	3.8 [2.9-4.9]	19.6 [-8.2 – 57.4]	0.35	3.6 [3.3-3.9]	3.7 [2.5-4.8]	-2.2 [-5.4 – 31.4]	0.61	0.53
Ea/Ees	0.70 [0.63-0.87]	0.67 [0.54-0.57]	-18.0 [-27.4 – 15.2]	0.35	0.70 [0.44-0.85]	0.57 [0.56-0.77]	2.7 [-15.5 – 29.3]	0.87	0.53

A, atrial contraction transmitral flow velocity at pulsed wave Doppler; AE, time between end of atrial contraction and mitral valve closure and mitral opening with initiation of early filling; DBP, diastolic blood pressure; DT, transmitral early flow velocity deceleration time; E, early transmitral velocity at pulsed wave Doppler; E’, tissue Doppler velocity of the mitral annulus averaged between medial and lateral; Ea, arterial elastance; EES, end-systolic elastance; ET, ejection time; HR, heart rate; LVEDV, left ventricular end-diastolic volume; LVEF, left ventricular ejection fraction; LVESV, left ventricle end-systolic volume; SBP, systolic blood pressure. Data are presented as median and interquartile range. Bold font indicates statistical significance.

### Quality of life 

Overall, at end-of-study improvements were observed across the ThyPRO-39 domains both in the LT4/placebo and in the LT4/LT3 groups. No significant differences were observed between the two groups. The data are ported in **Table 6**.

**Table 6:** Quality of life (Thy-PRO-39).

	LT4/LT3				LT4/placebo				
Domain	Baseline	End of study	Delta	p	Baseline	End of study	Delta	p	p for Delta
Goiter	60.0±41.0	15.0±25.3	-45.0±38.0	0.057	23.8±36.8	0.0±0.0	-23.8±36.8	0.137	0.355
Hyperthyroid	25.0±20.7	10.0±19.0	-15.0±18.1	0.136	23.2±29.9	10.7±10.6	-12.5±20.4	0.156	0.829
Hypothyroid	36.2±34.3	26.2±27.4	-10.0±24.0	0.405	11.6±17.5	14.3±14.8	2.7±17.2	0.695	0.310
Eye	26.7±27.2	21.7±29.8	-5.0±12.6	0.426	16.7±17.3	3.6±17.3	-13.1±17.9	0.101	0.408
Tiredness	78.3±31.5	41.7±40.4	-36.7±45.1	0.143	45.8±28.9	34.5±16.3	-11.2±19.3	0.174	0.207
Cognitive	60.0±42.2	26.7±29.7	-33.3±45.3	0.175	23.8±15.6	9.5±8.9	-14.3±15.8	0.054	0.320
Anxiety	65.0±38.4	23.3±21.6	-41.7±45.3	0.109	19.1±15.7	4.8±6.6	-14.3±19.1	0.095	0.178
Depressivity	56.7±42.2	26.7±30.8	-30.0±42.7	0.192	16.6±12.7	14.3±6.3	-2.3±11.5	0.609	0.128
Emotional	61.7±32.0	21.7±20.9	-40.0±41.8	0.099	21.4±22.5	11.9±11.6	-9.5±19.0	0.231	0.116
Social life	25.0±34.3	0.0±0.0	-25.0±34.3	0.179	9.5±14.0	0.0±0.0	-9.5±14.0	0.121	0.301
Daily life	50.0±46.8	11.7±18.3	-38.3±51.2	0.211	9.5±14.0	2.4±4.0	-7.1±13.1	0.199	0.148
Appearance	50.0±47.1	11.7±16.2	-38.3±62.8	0.244	17.9±37.4	8.3±22.0	-9.5±47.2	0.613	0.384
Composite	65.0±37.9	20.0±27.4	-45.0±64.7	0.195	17.9±23.8	3.6±9.4	-14.3±28.3	0.231	0.285

ThyPRO-39 score: 0-100±SD, higher value worst outcome. No correction was made for multiple comparisons.

## Discussion

Since the seminal observations of Dr. Morreale d’Escobar (5, 6) demonstrating that in animal models of hypothyroidism only LT4/LT3 combination therapy could restore T3 and T4 concentrations in most tissues, several trials have attempted to assess whether humans would benefit from such a regimen (12–25). The results have been conflicting, although a plurality of participants preferred T3-containing therapy regimens, mostly driven by reported weight loss (12–16). Heterogeneity of the study populations, with the inclusion of participants requiring low-dose LT4, thus presumably with residual endogenous TH production was recognized as a major confounder (26). Our study was unique because we characterized patients prior to total thyroidectomy, enabling the comparison of the two thyroid replacement regimens against the baseline of endogenous euthyroidism. Prior studies have evaluated TH levels pre- and post-thyroidectomy (8, 42), but none has attempted to characterize in detail the metabolic profile of patients by performing dense phenotyping.

The LT4/placebo group data provide empirical confirmation that the initial LT4 dose of 1.6 mcg dose (1) is accurate, the minimal reduction in dose when compared to our original observation (32, 40) can be attributed to the trice daily regimen adopted in that study, which could have led to adherence problems. Conversely, most of the patients in the LT4/LT3 group required a significant decrease in their LT4 dose, suggesting that our estimation of the dose adjustment (33) was not sufficient.

Our study demonstrated clear differences in TH concentrations between the two regimens following thyroidectomy. The LT4/placebo group data demonstrated that LT4 alone does not normalize circulating TH concentrations (9), while LT4/LT3 combination therapy may. Of interest, our data appear in contrast with Dr. Jonklaas’ report which did not demonstrate significant changes in T3 levels in patients undergoing thyroidectomy. It is worth noting that several patients with benign thyroid pathology underwent subtotal thyroidectomy, with the potential confounder of residual thyroid hormone production. Conversely, patients with malignant disease had lower TSH when compared to their pre-surgical baseline (8). These factors may have played a role in the apparent discrepancies between the observations. We did not measure trough total T3 levels, and most study participants underwent phlebotomy 1-3 hour following LT3 administration, which approximates the Tmax (33); hence the increase in serum total T3 observed at end-of-study in the LT4/LT3 group likely represents an overestimation.

Following thyroidectomy the LT4/placebo group experienced a non-significant weight gain whose effect size is consistent with the observations reported in a recent meta-analysis (43), while LT4/LT3 therapy appears to prevent it. Interestingly, the whole room indirect calorimetry data indicate divergent trends in energy expenditure between the LT4/placebo and the LT4/LT3 groups. Projected over one year, a decrease of 39.5 calorie/day observed in the LT4/placebo group would correspond to approximately 2 Kg of weight gain, which could be counterbalanced with the equivalent of ten days of fasting. These latter estimates are likely overstated since they do not take in account compensatory mechanisms (44).

Similar to the weight data, the LT4/placebo group experienced non-significant increase in total- and LDL-cholesterol which did not occur in the LT4/LT3 group, again consistent with the observation that LT4 alone does not restore euthyroidism.

The LT4/LT3 group had a small yet statistically significant improvement in Tei index, a measure cardiac performance (38). Interestingly, this is consistent with our LT3 vs. LT4 therapy trial where a marginal improvement in diastolic function was observed in the LT3-treated arm (32).

Overall, both groups showed a trend toward improvement in quality of life when compared to baseline (pre-surgery). It is possible that the anxiety associated with the upcoming surgery may have played a role. Indeed, the changes in “eye” domain which one would not expect be affected by the surgery (since Graves’ disease and thyrotoxicosis were exclusion criteria) support this interpretation. This is consistent with the observations of Azaria and colleagues (45).

The study was conducted during the COVID-19 pandemic which hampered recruitment and retention, as many potential participants objected to the “clinically unnecessary” pre-surgical and subsequent overnight admissions for baseline energy expenditure recording and overnight follow up studies. This led to an unanticipated limited number of participants and a significant attrition rate, causing an underpowered study and the need to use suboptimal (last measure carried forward in individuals who did not complete the 6-months visit) statistical analysis. It should be noted that the primary goal of the study was to provide point estimates for the design of larger intervention studies (30). To this end, the trend and the consistency of the findings clearly provides an unequivocal “go” to proceed with larger studies. By applying the point estimates of this study in *post-hoc* analyses, 11 participants in each treatment arm would provide 80% power to demonstrate a difference in weight, while 16 participants would provide 80% power to demonstrate a difference in total cholesterol between groups similar to the ones we observed in our study.

Another limitation of the study is that by design the recruitment was limited to patients undergoing total thyroidectomy, thus resulting in hypothyroidism devoid of residual TH production. It is possible that the point estimates obtained in this population comparing LT4/LT3 therapy to LT4/placebo are larger than the effects in patients with hypothyroidism due to autoimmune thyroid disease who presumably have some degree of residual TH production (26).

Our study design did not allow for adjustment of LT3 dosing neither as ratio to LT4 nor to participants’ weight. Within the LT4/LT3 treatment group the ratio LT3/LT4 ranged between 1:10 and 1:6, above the estimated endogenous T3 production from the thyroid gland (46). This is an obvious limitation driven by the practical need to use commercially available LT3 formulations which could then be utilized in larger studies. The trend toward a decrease in TSH, associated with decrease in weight and lipids observed in the LT4/LT3 group compared to baseline, suggests that the LT3 dosing employed in this study is supraphysiologic (pharmacologic). This is an important consideration which will need to be addressed by subsequent studies. Prior studies demonstrated that changes in TSH achieved by modulation of LT4 dose did not result in changes in indices of TH action (weight, cholesterol, energy expenditure) (47), thus the differences observed between groups are unlikely attributable to the non-significant differences in TSH at the end of the study. Of note, the “low” TSH in that particular study was higher than the average TSH observed in the LT4/LT3 group, hence one could speculate that the LT4 dose was not sufficient to exert a measurable metabolic effect. We did not measure additional indices of TH action such as sex hormone binding globulin or angiotensin converting enzyme (48). In the context of a feasibility study for a larger (effectiveness) trial these assays would have provided only limited additional information. Due to the limited number of patients, an assessment of the role of common polymorphisms in the type-2 deiodinase and TH transporter genes (49–51) on the response to therapy were not feasible.

Despite its limitations, this study has clearly demonstrated that LT4 alone, while normalizing TSH, does not restore euthyroidism when considering the circulating TH levels (9). Moreover, the lipid profile and weight data, which are clinically relevant indices of TH action, suggest that “optimal” LT4 therapy (1) in individuals devoid of endogenous TH production not only results in measurable abnormalities in circulating TH homeostasis, but possibly in inability of restoring euthyroidism at important end-organ targets of the hormonal action. Conversely, the supplementation of LT3 appears to be able to prevent these changes. The consistency of trends across multiple indices of TH action does not support the interpretation that our findings are due to type II error.

In conclusion, this feasibility study provides supportive evidence to design adequately powered large studies to evaluate the efficacy and effectiveness of LT4/LT3 combination therapy for the treatment of hypothyroidism, at least in patients undergoing total thyroidectomy.

## Data Availability

The raw data supporting the conclusions of this article will be made available by the authors, without undue reservation.

## References

[1] Jonklaas J Bianco AC Bauer AJ Burman KD Cappola AR Celi FS. Guidelines for the treatment of hypothyroidism: prepared by the american thyroid association task force on thyroid hormone replacement. Thyroid. (2014) 24:1670–751. doi: 10.1089/thy.2014.0028 PMC4267409 25266247

[2] Saravanan P Chau WF Roberts N Vedhara K Greenwood R Dayan CM. Psychological well-being in patients on ‘adequate’ doses of l-thyroxine: results of a large, controlled community-based questionnaire study. Clin Endocrinol (Oxf). (2002) 57:577–85. doi: 10.1046/j.1365-2265.2002.01654.x 12390330

[3] Ettleson MD Prieto WH Russo PST de Sa J Wan W Laiteerapong N. Serum thyrotropin and triiodothyronine levels in levothyroxine-treated patients. J Clin Endocrinol Metab. (2023) 108:e258–e66. doi: 10.1210/clinem/dgac725 PMC10413428 36515655

[4] Batistuzzo A Salas-Lucia F Gereben B Ribeiro MO Bianco AC. Sustained pituitary T3 production explains the T4-mediated TSH feedback mechanism. Endocrinology. (2023) 164. doi: 10.1210/endocr/bqad155 PMC10637099 37864846

[5] Escobar-Morreale HF Obregon MJ Escobar del Rey F Morreale de Escobar G. Replacement therapy for hypothyroidism with thyroxine alone does not ensure euthyroidism in all tissues, as studied in thyroidectomized rats. J Clin Invest. (1995) 96:2828–38. doi: 10.1172/JCI118353 PMC185993 8675653

[6] Escobar-Morreale HF del Rey FE Obregon MJ de Escobar GM. Only the combined treatment with thyroxine and triiodothyronine ensures euthyroidism in all tissues of the thyroidectomized rat. Endocrinology. (1996) 137:2490–502. doi: 10.1210/endo.137.6.8641203 8641203

[7] Werneck de Castro JP Fonseca TL Ueta CB McAninch EA Abdalla S Wittmann G. Differences in hypothalamic type 2 deiodinase ubiquitination explain localized sensitivity to thyroxine. J Clin Invest. (2015) 125:769–81. doi: 10.1172/JCI77588 PMC4319436 25555216

[8] Jonklaas J Davidson B Bhagat S Soldin SJ. Triiodothyronine levels in athyreotic individuals during levothyroxine therapy. JAMA. (2008) 299:769–77. doi: 10.1001/jama.299.7.769 18285588

[9] Gullo D Latina A Frasca F Le Moli R Pellegriti G Vigneri R. Levothyroxine monotherapy cannot guarantee euthyroidism in all athyreotic patients. PloS One. (2011) 6:e22552. doi: 10.1371/journal.pone.0022552 21829633 PMC3148220

[10] Stock JM Surks MI Oppenheimer JH. Replacement dosage of L-thyroxine in hypothyroidism. A re-evaluation. N Engl J Med. (1974) 290:529–33. doi: 10.1056/NEJM197403072901001 4811096

[11] Penna GC Bensenor IM Bianco AC Ettleson MD. Thyroid hormone homeostasis in levothyroxine-treated patients: findings from ELSA-brasil. J Clin Endocrinol Metab. (2024) 109:2504–12. doi: 10.1210/clinem/dgae139 PMC11403308 38506164

[12] Bunevicius R Kazanavicius G Zalinkevicius R Prange AJ Jr. Effects of thyroxine as compared with thyroxine plus triiodothyronine in patients with hypothyroidism. N Engl J Med. (1999) 340:424–9. doi: 10.1056/NEJM199902113400603 9971866

[13] Escobar-Morreale HF Botella-Carretero JI Gomez-Bueno M Galan JM Barrios V Sancho J. Thyroid hormone replacement therapy in primary hypothyroidism: a randomized trial comparing L-thyroxine plus liothyronine with L-thyroxine alone. Ann Intern Med. (2005) 142:412–24. doi: 10.7326/0003-4819-142-6-200503150-00007 15767619

[14] Nygaard B Jensen EW Kvetny J Jarlov A Faber J. Effect of combination therapy with thyroxine (T4) and 3,5,3’-triiodothyronine versus T4 monotherapy in patients with hypothyroidism, a double-blind, randomised cross-over study. Eur J Endocrinol. (2009) 161:895–902. doi: 10.1530/EJE-09-0542 19666698

[15] Bunevicius R Jakuboniene N Jurkevicius R Cernicat J Lasas L Prange AJ Jr. Thyroxine vs thyroxine plus triiodothyronine in treatment of hypothyroidism after thyroidectomy for Graves’ disease. Endocrine. (2002) 18:129–33. doi: 10.1385/ENDO:18:2:129 12374459

[16] Shakir MKM Brooks DI McAninch EA Fonseca TL Mai VQ Bianco AC. Comparative effectiveness of levothyroxine, desiccated thyroid extract, and levothyroxine+Liothyronine in hypothyroidism. J Clin Endocrinol Metab. (2021) 106:e4400–e13. doi: 10.1210/clinem/dgab478 PMC8530721 34185829

[17] Rodriguez T Lavis VR Meininger JC Kapadia AS Stafford LF. Substitution of liothyronine at a 1:5 ratio for a portion of levothyroxine: effect on fatigue, symptoms of depression, and working memory versus treatment with levothyroxine alone. Endocr Pract. (2005) 11:223–33. doi: 10.4158/EP.11.4.223 PMC1455482 16006298

[18] Walsh JP Shiels L Lim EM Bhagat CI Ward LC Stuckey BG. Combined thyroxine/liothyronine treatment does not improve well-being, quality of life, or cognitive function compared to thyroxine alone: a randomized controlled trial in patients with primary hypothyroidism. J Clin Endocrinol Metab. (2003) 88:4543–50. doi: 10.1210/jc.2003-030249 14557419

[19] Appelhof BC Fliers E Wekking EM Schene AH Huyser J Tijssen JG. Combined therapy with levothyroxine and liothyronine in two ratios, compared with levothyroxine monotherapy in primary hypothyroidism: a double-blind, randomized, controlled clinical trial. J Clin Endocrinol Metab. (2005) 90:2666–74. doi: 10.1210/jc.2004-2111 15705921

[20] Sawka AM Gerstein HC Marriott MJ MacQueen GM Joffe RT. Does a combination regimen of thyroxine (T4) and 3,5,3’-triiodothyronine improve depressive symptoms better than T4 alone in patients with hypothyroidism? Results of a double-blind, randomized, controlled trial. J Clin Endocrinol Metab. (2003) 88:4551–5. doi: 10.1210/jc.2003-030139 14557420

[21] Clyde PW Harari AE Getka EJ Shakir KM. Combined levothyroxine plus liothyronine compared with levothyroxine alone in primary hypothyroidism: a randomized controlled trial. JAMA. (2003) 290:2952–8. doi: 10.1001/jama.290.22.2952 14665656

[22] Fadeyev VV Morgunova TB Melnichenko GA Dedov II. Combined therapy with L-thyroxine and L-triiodothyronine compared to L-thyroxine alone in the treatment of primary hypothyroidism. Hormones (Athens). (2010) 9:245–52. doi: 10.14310/horm.2002.1274 20688622

[23] Saravanan P Simmons DJ Greenwood R Peters TJ Dayan CM. Partial substitution of thyroxine (T4) with tri-iodothyronine in patients on T4 replacement therapy: results of a large community-based randomized controlled trial. J Clin Endocrinol Metab. (2005) 90:805–12. doi: 10.1210/jc.2004-1672 15585551

[24] Siegmund W Spieker K Weike AI Giessmann T Modess C Dabers T. Replacement therapy with levothyroxine plus triiodothyronine (bioavailable molar ratio 14: 1) is not superior to thyroxine alone to improve well-being and cognitive performance in hypothyroidism. Clin Endocrinol (Oxf). (2004) 60:750–7. doi: 10.1111/j.1365-2265.2004.02050.x 15163340

[25] Valizadeh M Seyyed-Majidi MR Hajibeigloo H Momtazi S Musavinasab N Hayatbakhsh MR. Efficacy of combined levothyroxine and liothyronine as compared with levothyroxine monotherapy in primary hypothyroidism: a randomized controlled trial. Endocr Res. (2009) 34:80–9. doi: 10.1080/07435800903156340 19701833

[26] Madan R Celi FS. Combination therapy for hypothyroidism: rationale, therapeutic goals, and design. Front Endocrinol (Lausanne). (2020) 11:371. doi: 10.3389/fendo.2020.00371 32733377 PMC7360670

[27] Garber JR Cobin RH Gharib H Hennessey JV Klein I Mechanick JI. Clinical practice guidelines for hypothyroidism in adults: cosponsored by the American Association of Clinical Endocrinologists and the American Thyroid Association. Thyroid. (2012) 22:1200–35. doi: 10.1089/thy.2012.0205 22954017

[28] Guglielmi R Frasoldati A Zini M Grimaldi F Gharib H Garber JR. Italian association of clinical endocrinologists statement-replacement therapy for primary hypothyroidism: A brief guide for clinical practice. Endocr Pract. (2016) 22:1319–26. doi: 10.4158/EP161308.OR 27482609

[29] Brenta G Vaisman M Sgarbi JA Bergoglio LM Andrada NC Bravo PP. Clinical practice guidelines for the management of hypothyroidism. Arq Bras Endocrinol Metabol. (2013) 57:265–91. doi: 10.1590/S0004-27302013000400003 23828433

[30] Jonklaas J Bianco AC Cappola AR Celi FS Fliers E Heuer H. Evidence-based use of levothyroxine/liothyronine combinations in treating hypothyroidism: A consensus document. Thyroid. (2021) 31:156–82. doi: 10.1089/thy.2020.0720 PMC8035928 33276704

[31] Burch HB. Drug effects on the thyroid. N Engl J Med. (2019) 381:749–61. doi: 10.1056/NEJMra1901214 31433922

[32] Celi FS Zemskova M Linderman JD Smith S Drinkard B Sachdev V. Metabolic effects of liothyronine therapy in hypothyroidism: a randomized, double-blind, crossover trial of liothyronine versus levothyroxine. J Clin Endocrinol Metab. (2011) 96:3466–74. doi: 10.1210/jc.2011-1329 PMC3205882 21865366

[33] Van Tassell B Wohlford G Linderman JD Smith S Yavuz S Pucino F. Pharmacokinetics of L-triiodothyronine in patients undergoing thyroid hormone therapy withdrawal. Thyroid. (2019) 29:1371–9. doi: 10.1089/thy.2019.0101 PMC6797066 31364488

[34] Watt T Bjorner JB Groenvold M Rasmussen AK Bonnema SJ Hegedus L. Establishing construct validity for the thyroid-specific patient reported outcome measure (ThyPRO): an initial examination. Qual Life Res. (2009) 18:483–96. doi: 10.1007/s11136-009-9460-8 19288224

[35] Chen S Wohlers E Ruud E Moon J Ni B Celi FS. Improving temporal accuracy of human metabolic chambers for dynamic metabolic studies. PloS One. (2018) 13:e0193467. doi: 10.1371/journal.pone.0193467 29689096 PMC5916490

[36] Chen S Scott C Pearce JV Farrar JS Evans RK Celi FS. An appraisal of whole-room indirect calorimeters and a metabolic cart for measuring resting and active metabolic rates. Sci Rep. (2020) 10:14343. doi: 10.1038/s41598-020-71001-1 32868770 PMC7459349

[37] Mitchell C Rahko PS Blauwet LA Canaday B Finstuen JA Foster MC. Guidelines for performing a comprehensive transthoracic echocardiographic examination in adults: recommendations from the American society of echocardiography. J Am Soc Echocardiogr. (2019) 32:1–64. doi: 10.1016/j.echo.2018.06.004 30282592

[38] Tei C Ling LH Hodge DO Bailey KR Oh JK Rodeheffer RJ. New index of combined systolic and diastolic myocardial performance: a simple and reproducible measure of cardiac function–a study in normals and dilated cardiomyopathy. J Cardiol. (1995) 26:357–66. 8558414

[39] Chantler PD Lakatta EG Najjar SS. Arterial-ventricular coupling: mechanistic insights into cardiovascular performance at rest and during exercise. J Appl Physiol (1985). (2008) 105:1342–51. doi: 10.1152/japplphysiol.90600.2008 PMC2576043 18617626

[40] Celi FS Zemskova M Linderman JD Babar NI Skarulis MC Csako G. The pharmacodynamic equivalence of levothyroxine and liothyronine: a randomized, double blind, cross-over study in thyroidectomized patients. Clin Endocrinol (Oxf). (2010) 72:709–15. doi: 10.1111/j.1365-2265.2009.03700.x PMC2888764 20447070

[41] Hauge C Breitschaft A Hartoft-Nielsen ML Jensen S Baekdal TA. Effect of oral semaglutide on the pharmacokinetics of thyroxine after dosing of levothyroxine and the influence of co-administered tablets on the pharmacokinetics of oral semaglutide in healthy subjects: an open-label, one-sequence crossover, single-center, multiple-dose, two-part trial. Expert Opin Drug Metab Toxicol. (2021) 17:1139–48. doi: 10.1080/17425255.2021.1955856 34289755

[42] Ito M Miyauchi A Hisakado M Yoshioka W Kudo T Nishihara E. Thyroid function related symptoms during levothyroxine monotherapy in athyreotic patients. Endocr J. (2019) 66:953–60. doi: 10.1507/endocrj.EJ19-0094 31270299

[43] Huynh CN Pearce JV Kang L Celi FS. Weight gain after thyroidectomy: A systematic review and meta-analysis. J Clin Endocrinol Metab. (2021) 106:282–91. doi: 10.1210/clinem/dgaa754 PMC7765639 33106852

[44] Hall KD Sacks G Chandramohan D Chow CC Wang YC Gortmaker SL. Quantification of the effect of energy imbalance on bodyweight. Lancet. (2011) 378:826–37. doi: 10.1016/S0140-6736(11)60812-X PMC3880593 21872751

[45] Azaria S Cherian AJ Gowri M Thomas S Gaikwad P Mj P. Impact of thyroidectomy on quality of life in benign goitres: results from a prospective cohort study. Langenbecks Arch Surg. (2022) 407:1193–9. doi: 10.1007/s00423-021-02391-7 34988642

[46] Pilo A Iervasi G Vitek F Ferdeghini M Cazzuola F Bianchi R. Thyroidal and peripheral production of 3,5,3’-triiodothyronine in humans by multicompartmental analysis. Am J Physiol. (1990) 258:E715–26. doi: 10.1152/ajpendo.1990.258.4.E715 2333963

[47] Samuels MH Kolobova I Niederhausen M Purnell JQ Schuff KG. Effects of altering levothyroxine dose on energy expenditure and body composition in subjects treated with LT4. J Clin Endocrinol Metab. (2018) 103:4163–75. doi: 10.1210/jc.2018-01203 PMC6194808 30165520

[48] Jansen HI Bruinstroop E Heijboer AC Boelen A. Biomarkers indicating tissue thyroid hormone status: ready to be implemented yet? J Endocrinol. (2022) 253:R21–45. doi: 10.1530/JOE-21-0364 35256536

[49] Mentuccia D Proietti-Pannunzi L Tanner K Bacci V Pollin TI Poehlman ET. Association between a novel variant of the human type 2 deiodinase gene Thr92Ala and insulin resistance: evidence of interaction with the Trp64Arg variant of the beta-3-adrenergic receptor. Diabetes. (2002) 51:880–3. doi: 10.2337/diabetes.51.3.880 11872697

[50] Peeters RP van den Beld AW Attalki H Toor H de Rijke YB Kuiper GG. A new polymorphism in the type II deiodinase gene is associated with circulating thyroid hormone parameters. Am J Physiol Endocrinol Metab. (2005) 289:E75–81. doi: 10.1152/ajpendo.00571.2004 15727947

[51] Sterenborg R Galesloot TE Teumer A Netea-Maier RT Speed D Meima ME. The effects of common genetic variation in 96 genes involved in thyroid hormone regulation on TSH and FT4 concentrations. J Clin Endocrinol Metab. (2022) 107:e2276–e83. doi: 10.1210/clinem/dgac136 PMC9315164 35262175

